# A rare landmark of cecal intubation 

**Published:** 2019

**Authors:** Ahmed T. Chatila, Mohammad Bilal, Praveen Guturu

**Affiliations:** 1 *Department of Internal Medicine, University of Texas Medical Branch, Galveston, TX, USA*; 2 *Department of Gastroenterology & Hepatology, University of Texas Medical Branch, Galveston TX, USA *

**Question**

**Figure 1:**
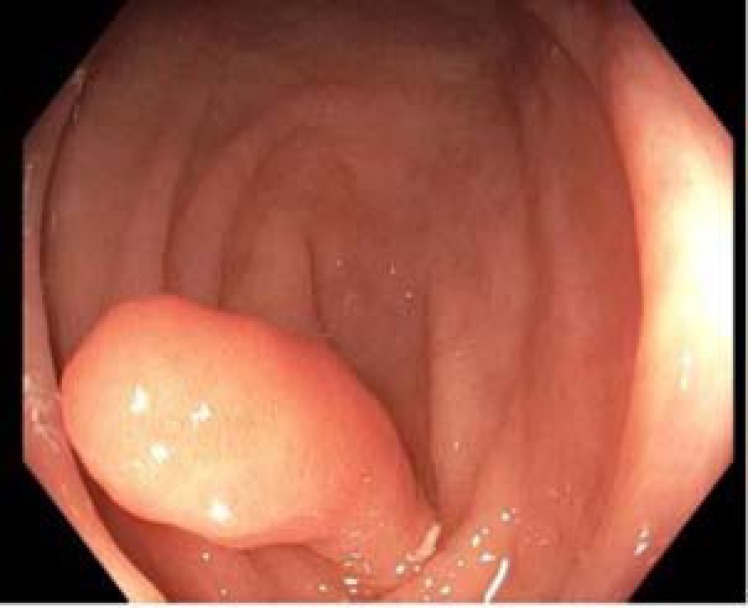
Invaginated appendix seen on colonoscopy

**Figure 2:**
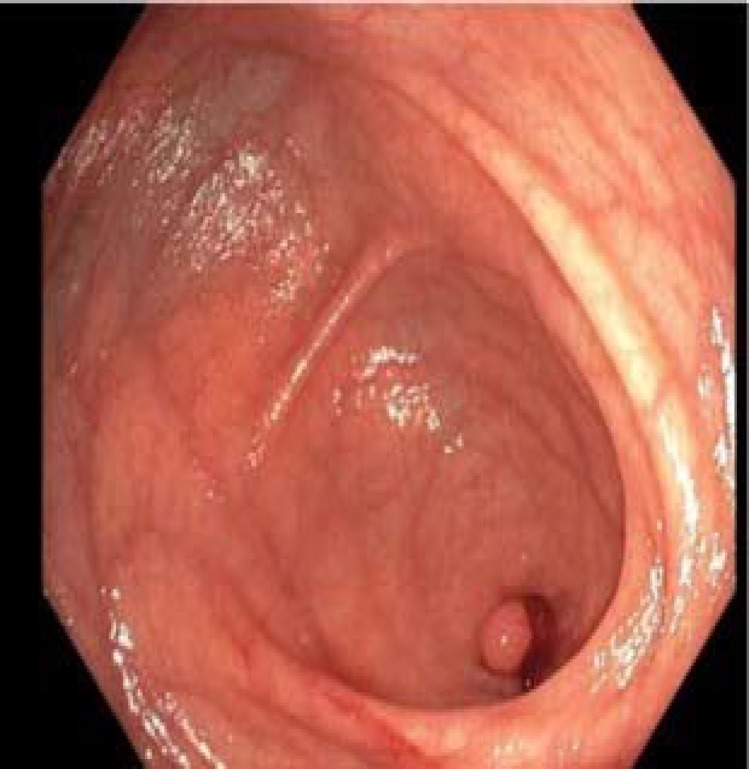
Invaginated appendix seen on colonoscopy

 A 51-year-old Caucasian female with a past history of hypertension and hypothyroidism presented for a screening colonoscopy. Her surgical history was significant for a total abdominal hysterectomy. Abdominal examination was normal and laboratory findings were unremarkable. CT scan of the abdomen was performed which did not show any abnormalities or pathologies upon read. The patient underwent colonoscopy demonstrating diverticulosis and a 6 mm pedunculated polyp in the sigmoid colon. Polypectomy was performed using hot snare. A polypoid lesion was seen at the base of the appendiceal orifice (Figure 1-2).

**What is the diagnosis?**

**How should it be managed?**

**Answer**

The colonoscopy demonstrated a pedunculated lesion consistent with an invaginated appendix. The lesion was filiform with a normal appearing mucosa on endoscopic imagining with superficial biopsies of the lesion later displaying no dysplastic changes. 

An invaginated appendix occurs when the appendix pulls itself into the cecum (1). The pathophysiological mechanisms can be divided into two groups: anatomical and pathological. Anatomical conditions include fetal abnormalities, a wide appendicular lumen, a meso-appendix free from fat, a mobile appendicular wall, and an appendix unfixed by peritoneal folds. Pathological conditions resulting in an invaginated appendix include irritation secondary to fecaliths, foreign bodies, malignancy, parasites, endometrial implants, and lymphoid follicles (2). It is important for gastroenterologists to be aware of this condition to avoid unnecessary intervention and to consider this finding as a risk factor for appendiceal intussusception. 
